# A Clinical Guidance for the Management of Patients With Hepatoid Adenocarcinoma and A Case Series

**DOI:** 10.1002/cam4.71398

**Published:** 2026-02-05

**Authors:** Christina Liava, Sudhakar Venkatesh, Michael S. Torbenson, Patrick S. Kamath, Moira Hilscher

**Affiliations:** ^1^ Division of Gastroenterology and Hepatology Mayo Clinic College of Medicine and Science Rochester Minnesota USA; ^2^ Radiology Department Mayo Clinic College of Medicine and Science Rochester Minnesota USA; ^3^ Division of Anatomic Pathology, Department of Laboratory Medicine and Pathology Mayo Clinic College of Medicine and Science Rochester Minnesota USA

**Keywords:** case series, clinical guidance, hepatoid adenocarcinoma, molecular biomarkers

## Abstract

Hepatoid adenocarcinoma (HAC) is a rare extrahepatic tumor of non‐germ cell origin that morphologically resembles hepatocellular carcinoma (HCC). HAC has a propensity to metastasize to the liver and therefore may be mistaken for HCC. There is a lack of standardized treatment protocols, and further studies are needed to evaluate the benefit of targeted therapy and immunotherapy. Recent studies have reported that tumor protein 53 (*TP53*) gene mutations are associated with increased expression of programmed cell death ligand‐1 (PD‐L1), which may be a predictor of response to PD‐L1 targeted checkpoint inhibitors.

This review provides a clinical guidance for the management of patients with HAC by summarizing the salient clinical features, risk factors, diagnostic criteria, differential diagnosis, new therapeutic approaches, and prognosis of this rare tumor. Furthermore, we reviewed the Mayo Clinic experience to describe the clinical characteristics of 15 patients diagnosed with HAC.

HAC is usually diagnosed at an advanced stage with distant metastases. In patients diagnosed with liver lesions that have similar radiologic and histologic features to HCC, particularly in the absence of underlying chronic liver disease, further evaluation should be performed to rule out HAC. Communication between medical subspecialties is important to avoid misdiagnosis and prevent further disease progression. In our patient cohort *TP53* was the most frequently mutated gene (5 out of 8, 62.5%) and PD‐L1 expression showed a positive score in 3 out of 6 patients (50%). However, only a few patients received immunotherapy (6 out of 14, 42.9%) suggesting that the numbers are too small to draw a conclusion about its efficacy in treating HAC.

## 1 Introduction

Hepatoid adenocarcinoma (HAC) is a rare extrahepatic tumor of non‐germ cell origin which morphologically resembles hepatocellular carcinoma (HCC) and commonly produces alpha fetoprotein (AFP) [1, 2, 3]. It represents a distinct type of AFP‐producing tumors, of which HCC is the most prevalent; AFP‐producing gastric cancers and yolk sac tumor‐like carcinomas are also AFP‐producing tumors, in addition to other less common carcinomas from different organs [4]. HAC has a propensity to metastasize to the liver and therefore may be mistaken for HCC [4]. The first case of HAC was reported in 1970 and was called AFP‐producing gastric adenocarcinoma [1]. In 1985, Ishimura et al. used the term HAC to describe this unique type of cancer [3]. HAC has been reported in several different primary organ sites, including the stomach, esophagus, lung, colon, pancreas, gallbladder, small intestine, bladder, ureter, uterus, testis and ovary [5, 6, 7]. At these extrahepatic sites, the tumor may be mistaken for metastatic HCC. HAC is characterized by an aggressive clinical course and often portends a poor prognosis [4].

A majority of the scientific literature on HAC consists of case reports or limited patient series. As a result, the accurate identification, diagnosis, and management of this rare tumor remain challenging. Furthermore, there is a lack of data that summarizes the existing evidence about the clinical characteristics and management of patients diagnosed with HAC. The aim of this review is to provide clinical guidance for the management of patients with HAC by summarizing the salient clinical features, risk factors, diagnostic criteria, differential diagnosis, new therapeutic approaches, and prognosis of this rare tumor. Furthermore, we reviewed the Mayo Clinic experience to describe the clinical characteristics of 15 patients diagnosed with HAC, their molecular features, clinical application of immune‐based therapies, comparison of treatment efficacy, and prognostic factors.

## 2 Epidemiology, Clinical Features, and Risk Factors

Most case reports of HAC originate from Asia, particularly China and Japan [4]. The overall incidence of HAC between 2000 and 2016 was 0.014 per 100,000 people in China, based on findings of the most recent systematic review and meta‐analysis [8]. The most common primary site in this study was the lung (49.6%), in contrast to previously published data, in which the stomach was the most common site. Most of the patients were elderly, of whom 66.1% were over 60 years old. On the other hand, a literature review of 217 patients with HAC published in 2013 found that the most common primary site of HAC was the stomach (83.9%), followed by the gallbladder (3.7%), uterus (3.2%), lung (2.3%), and urinary bladder (1.8%) [5]. HAC originating from the esophagus and peritoneum was very rare (0.9%). HACs of the rectum, colon, testis, ovary, jejunum, fallopian tube, kidney, thymus, ureter or adrenal glands were only reported in single‐case reports (each < 0.5%) [5, 9]. Another literature review of 261 HAC cases published in 2010, found similarly that the stomach was the most common primary site (63%), followed by the ovaries (10%), lung (5%), and pancreas (4%) [9]. Apart from the gynecological HAC cases and those originating from the gallbladder, in other primary sites a male predominance was observed (male to female ratio 2.4:1) with a median age over 60 years [5, 9]. The distinct features of HACs based on primary tumor location are summarized below.

### 2.1 Stomach

Hepatoid adenocarcinoma of the stomach (HAS) is a rare type of gastric cancer, accounting for 0.3%–1% of all gastric cancer with an incidence of 0.58–0.83 cases per million people [10, 11, 12]. HAS is more commonly diagnosed in elderly patients and has a strong male predominance with a reported male to female ratio of 11:4 [13]. The most common tumor location is the antrum (46%–55%), followed by the body (27%–31%), cardia (7%–21%), and other sites [14, 15]. The implication of Helicobacter pylori infection in the pathogenesis of HAS has not been established, unlike its proven link to the development of most gastric adenocarcinomas, particularly the intestinal type [16]. HAS often coexists with adenocarcinoma with hepatoid components localized in the invasive foci [2, 8, 12]. A molecular study has shown that hepatoid carcinoma cells were of identical origin to coexistent tubular adenocarcinomas [17]. The authors of this study hypothesized that intestinal tubular adenocarcinomas transdifferentiate to a hepatoid type adenocarcinoma over time and that this shift in subtype confers an enhanced metastatic capacity. Another study suggested that HAC of the stomach arises from an adenocarcinoma with intestinal phenotype [10].

The clinical symptoms of HAS are nonspecific, resembling the typical clinical manifestations of gastric cancer, such as abdominal pain, epigastric discomfort, gastrointestinal bleeding, and melena. A pooled cases series of 328 HAS cases found that the most frequent presenting symptom was abdominal pain (40.9%), followed by epigastric discomfort (23.5%), hematemesis and/or melena (18.3%), and weight loss (17.3%) [14]. The majority of HAS patients (80.6%) had elevated serum AFP levels. About 72.9% of HAS cases were poorly differentiated. Lymph node metastasis occurred in 78.4%, while vascular invasion was observed in 69.6%. Most HAS patients had distant metastasis preoperatively, particularly in the liver (78.5%), lungs (11.6%), peritoneum (6.6%), spleen (2.5%), and brain (0.8%). A literature review of 180 HAS cases from China found that the serum AFP levels of HAS patients without metastasis or liver involvement were significantly lower than those with metastasis or liver involvement [15].

### 2.2 Lung

Hepatoid adenocarcinoma of the lung (HAL) is a distinct form of lung adenocarcinoma that exhibits a low prevalence, with a male predominance accounting for approximately 80% of cases with the majority of them being smokers [18, 19, 20]. The primary tumor is mainly located in the upper lobe of the lung (68%–72%) [18] and especially the right lung (65.3%) [20]. Clinical symptoms of HAL are often non‐specific and similar to common lung cancer, and are related to the location and the size of the mass. A literature review of 49 HAL cases found that the most common symptom was cough (34.7%), followed by chest pain (16.3%), dyspnea (14.3%), and weight loss (12.2%). Of the 49 reported cases, only 26 had elevated AFP levels (53.1%) [20]. In this study, more than half of HAL cases underwent radical surgery of the mass and lymph nodes (59.2%) achieving a favorable prognosis. A previous literature review of 41 HAL cases found that 82% of patients had stage III or IV at first presentation [19].

### 2.3 Esophagus

HACs arising from the esophagus are extremely rare with a prevalence of 0.9% [5]. Only eight cases are reported in the literature [21, 22, 23, 24, 25, 26, 27, 28]. Esophageal HACs are more frequently observed in Asian populations with a male predominance. In all eight cases the tumor was located in the lower part of the esophagus in proximity to the gastroesophageal junction. An association between esophageal HAC and Barrett's esophagus has been described. However, this association is more prominent when HAC coexists with other histologic tumors, such as tubular adenocarcinoma and choriocarcinoma [21, 26], similar to HAS. Patients with esophageal HAC generally present with non‐specific symptoms including decreased appetite, weight loss, and fatigue. The tumor has an aggressive behavior and in the majority of patients, distant metastases are found at the time of first diagnosis. The most common distant metastasis site was the liver. Patients with liver metastases also tended to have elevated AFP levels [21].

### 2.4 Colon

Regarding HACs originating from the colon, a comprehensive literature review of 39 HAC colon cases revealed a male predominance with an age of 60 years or older. A history of ulcerative colitis was reported in six cases. The most frequent tumor site was the right colon (46.2%) [29]. In some case reports coexistence of colon HACs with adenocarcinomas was observed [30, 31]. The most commonly reported symptoms were blood in the stool (33.3%), followed by abdominal pain (28.2%), and other (38.5%), such as changes in bowel habits, urgency, and intestinal obstruction [29]. Almost half of patients with colon HACs had distant metastasis (48.7%) and the most frequent site was the liver (43.6%). Among the 32 cases, 27 had elevated AFP levels (84.4%) [29].

### 2.5 Small Intestine

Seven cases have been reported in the literature reporting HAC diagnosis in the jejunoileum [32], of whom two cases were accompanied by long‐standing Crohn's disease (CD) [33, 34], and six cases reported HAC in the duodenum [35, 36, 37, 38, 39, 40]. Both patients with HAC and CD had liver metastases and elevated AFP levels at presentation. The first patient presented with bowel obstruction and the second with abdominal pain, constipation, and nausea/vomiting [33, 34]. It is well established that patients with inflammatory bowel disease (IBD) are at an increased risk of developing adenocarcinomas of the gastrointestinal tract [41, 42].

### 2.6 Pancreas

The current World Health Organization (WHO) classification recognizes hepatoid morphology only as a possible variant of pancreatic ductal adenocarcinoma, which is associated with high rates of vascular invasion and early metastasis [43]. However, pancreatic neuroendocrine tumors may also show hepatoid differentiation; more specifically, the hepatoid variant, a morphological subtype that is associated with more aggressive clinical behavior [44]. Furthermore, a recent clinicopathological study demonstrated distinct histopathological and molecular features of two pancreatic tumors with hepatoid morphology. This fact illustrates that hepatoid differentiation may have been underestimated until now [45]. A literature review of 22 pancreatic HAC cases found that the mean patient age was 53 years old with a male predominance [46]. Most cases were asymptomatic or incidentally found (31.3%) on imaging studies. Of those patients who developed symptoms, the most common was abdominal pain (27.3%), followed by jaundice (18.2%), nausea/vomiting (18.2%), and weight loss (13.6%). Elevated AFP levels were found in 57.1%.

## 3 Diagnosis and Differential Diagnosis

### 3.1 Imaging

Computed tomography (CT) imaging of HAS may reveal thickening of the gastric walls with heterogeneous mild to moderate contrast enhancement, which likely represents areas of necrosis and hemorrhage within the tumor [47]. Interestingly, a recent study applied a CT‐based radiomics nomogram for distinguishing HAS from common gastric cancer (CGC) and achieved a high accuracy with an area under the curve (AUC) of 0.905 (95% CI: 0.796–0.968) in the test cohort [48]. Increased signal on diffusion‐weighted imaging has been observed in magnetic resonance images (MRI) of abdominal HAC [49]. HAC is often diagnosed in advanced stages and thus lymph node, liver, or distant metastases are frequently present at the time of diagnosis [50, 51]. The liver is the most common metastatic site of HAC. Similar to HCC, liver metastases of HAC often demonstrate arterial hyperattenuation due to dense blood vessels with late washout on CT imaging [52, 53], although atypical features such as retraction of the adjacent liver capsule have been described [54].

Liver metastases of HAC need to be differentiated from HCC, which often occurs in the setting of viral hepatitis, metabolic dysfunction‐associated steatotic liver disease, or cirrhosis. On the other hand, HAC tumors are usually not accompanied by chronic liver disease. Hemorrhage is a common feature of hepatic metastasis of HAC and HCC [53]. Most hepatic metastases of HAC show tumor necrosis regardless of tumor size [50, 55], while HCC necrosis occurs in lesions larger than 3 cm with a relatively low rate (10%–40%) [56]. There have been described two types of liver metastases in HAC: (1) large masses with adjacent portal vein cancer thrombus (portal vein cancer thrombus occurs in 60%–75%); and (2) many nodules of similar size that are not accompanied by portal vein cancer thrombus [50].

### 3.2 Serum Biomarkers

Depending on the primary tumor location, different frequencies of elevated serum biomarkers prior to HAC diagnosis have been reported in the literature, as summarized in Table 1. Studies have shown that the majority of patients with HAS have elevated levels of AFP; however, there is a subset of HAS that does not produce AFP [11, 47, 58, 59]. As a result, the elevation of serum AFP is not a justified criterion for the diagnosis of HAS. A subset of gastric adenocarcinomas produces AFP but lacks hepatoid features histologically and must be distinguished from HAS. Liu et al. [60] retrospectively analyzed 104 patients with gastric adenocarcinoma that had an elevated serum AFP and positive AFP staining of the primary lesion prior to gastrectomy. Of these patients, 45 had evidence of hepatoid differentiation and were diagnosed with HAS, and 59 were diagnosed with AFP‐producing gastric cancer (AFPGC) and were compared with stage‐matched CGC patients, as demonstrated in Table 2. No data is currently available to support the use of serum biomarkers for HCC, the glycoform of AFP, AFP‐L3, and the abnormal form of prothrombin, des‐γ‐carboxy prothrombin in the differentiation of HCC from HAC, as there is no evidence of their production in HAC.

**TABLE 1:** Frequency of elevated serum biomarkers based on HAC primary site [10, 18, 20, 29, 46, 51, 57].

HAC primary location	Alpha‐fetoprotein (AFP)	Carcinoembryonic antigen (CEA)	CA 19‐9
Stomach	80.6%	N/A	N/A
Lung	53%–76%	N/A	N/A
Esophagus	83.3%	N/A	N/A
Colon	84.4%	57.9%	42.9%
Pancreas	57.1%	47.6%	N/A

Abbreviation: HAC, hepatoid adenocarcinoma.

**TABLE 2:** Clinical differences between patients with HAS, CGC, and AFPGC [60].

Characteristics	Hepatoid adenocarcinoma of the stomach (HAS)	Common gastric cancer (CGC)	AFP‐producing gastric cancer (AFPGC)
AFP production	Higher probability	Lower probability	Higher probability
Vascular invasion	Higher probability	Lower probability	Lower probability
Liver metastases	Higher probability	Lower probability	Moderate probability
Time interval from gastrectomy to liver metastases	Shorter	Longer	Longer
1‐year survival rates	30%	95%	64%
2‐year survival rates	13%	57%	47%
5‐year survival rates	9%	38%	41%

Abbreviation: AFP, alpha‐fetoprotein.

The poor prognosis of HAC may relate to its expression of AFP. AFPGCs demonstrate high proliferative activity, enriched neovascularization, and significantly less apoptosis when compared with AFP‐negative gastric cancers [61]. Preoperative serum AFP levels ≥ 500 ng/mL are significantly associated with poorer overall survival and also correlate with poorer disease‐free survival in HAC patients [4]. Studies suggest that AFP may negatively impact lymphocyte transformation and thereby facilitate evasion of the host immune response [62]. AFP additionally has pro‐angiogenic and proliferative effects and can suppress apoptosis within tumors [63, 64, 65, 66, 67].

### 3.3 Histopathology

The gold standard for the diagnosis of HAC tumors is histopathology. HAC tumors often exhibit both hepatoid morphology and gland formation [4]. The hepatoid component of HAC may consist of tumor cells arranged in a trabecular pattern or solid nests of cells surrounded by fibrous stroma [60]. The tumor cells are often cuboidal or polygonal in shape with clear or eosinophilic cytoplasm, ovoid nuclei, and occasional hyaline globules. The adenocarcinoma components are typically intermingled with hepatoid areas [68]. Some studies suggest that the hepatoid features of HAC are more prominent in metastases detected within lymph nodes and the liver [12, 69, 70, 71], possibly due to enhanced proliferation of hepatoid tumor cells in these stromal environments [72].

### 3.4 Immunohistochemistry and Molecular Features

Definite diagnosis of HAC is difficult based on morphology findings alone. Further immunohistochemistry (IHC) analysis is needed for differential diagnosis. Liver metastases from HAC may be mistaken for HCC because of hepatoid histological findings and elevated serum AFP levels. Molecular marker expression can help to differentiate metastatic HAC from HCC (Table 3). IHC analysis reveals AFP staining in more than 90% of HACs regardless of the primary tumor site [5]. HACs also commonly stain positive for α‐1 antitrypsin, α‐1 chemotrypsin, caudal type homeobox 2 (CDX2), and mucin‐1 (MUC1) [5, 9]. Immunostaining markers of hepatocytic differentiation (such as hepatocyte paraffin 1 [HepPar1], Arginase, albumin in situ hybridization) are routinely positive in HAC [76], so careful correlation with imaging and clinical findings, histological morphology, and immunostaining findings is needed to make an accurate diagnosis. 

The hepatoid morphology and positive staining for markers of hepatocytic differentiation in HAC can lead to diagnostic challenges, primarily with a potential misdiagnosis of HCC. Important diagnostic clues include morphological features that are inconsistent with HCC, such as the presence of glands; these features are often focal, so may require careful histological examination. Additional important diagnostic clues are the immunohistochemical findings, and in many cases, HACs are only identified at this step. Here, the specific immunohistochemical findings will vary by tumor origin (e.g., stomach, colon, etc.), but the key feature is positivity for markers that are typically negative in HCC or findings that stain negative but are typically positive in HCC. HepPar1 is positive in the HCC component of the combined HCC with adenocarcinomatous components but is typically negative in HAC. Spalt‐like transcription factor 4 (SALL4), glypican‐3 (GPC3), AFP, cytokeratin 8 (CK8), CK18, and CK20 are often positive in HAC. Furthermore, mixed subtypes of combined hepatocellular cholangiocarcinoma have characteristic transition zones between the hepatocellular and glandular components as well as positive staining for CK7, in contrast to HAC [77, 78]. A third important diagnostic clue is the clinical and imaging findings. For example, evaluation of a liver mass that shows hepatoid morphology should always lead to consideration of the possibility of metastatic HAC in patients with known gastrointestinal mass lesions.

**TABLE 3:** Immunohistochemistry markers for distinguishing HAC from non‐hepatoid cancer types [5, 14, 29, 73, 74, 75].

Immunohistochemistry markers	Hepatoid adenocarcinoma—frequency of positivity	Non‐hepatoid cancers—frequency of positivity
Hep‐Par 1	High (65%–71%)	HCC (high), CGC (low), Liver metastasis from GI tract (low)
Alpha‐Fetoprotein (AFP)	High (92.1%)	HCC (moderate), CGC (very low)
Glypican‐3 (GPC‐3)	High (~100%)	HCC (high), CGC (very low)
Polyclonal CEA	High (75%–100%)	HCC (high), Liver metastases from GI tract (high)
Cytokeratin AE1/AE3	High (92.3%)	HCC (low)
Cytokeratin 18 (CK18)	High (~100%)	HCC (low)
Cytokeratin 19 (CK19)	High (~100%)	HCC (very low), CGC (high)
Cytokeratin 20 (CK20)	Low (25%)	HCC (low)
Cytokeratin 7 (CK7)	Low (15.4%)	HCC (low)
CD10	High (62.5%)	HCC (high), Liver metastasis from GI tract (low)
PLUNC	High (100%)	HCC (very low)
Arginase‐1	Low	HCC (high), Liver metastasis from GI tract (low)
Albumin	High	HCC (high), CGC (very low)
HER2	Moderate (31.3%)	HCC (low), CGC (moderate)

Abbreviations: CEA, carcinoembryonic antigen; CGC, common gastric cancer; GI, gastrointestinal; HCC, hepatocellular carcinoma; HER2, human epidermal growth factor receptor 2; PLUNC, palate, lung, nasal epithelial clone.

Regarding HAL tumors, the absence of distinctive features often leads to a misdiagnosis of HAL as other types of lung cancers or as a metastatic lung tumor from primary HCC [20]. Haninger et al. [6] proposed two criteria for establishing HAL diagnosis: (1) the tumor can be either pure HAC, or have features of typical acinar or papillary adenocarcinoma, signet‐ring cells or neuroendocrine carcinoma; and (2) AFP expression is not considered a prerequisite for diagnosis as long as other markers of hepatoid differentiation are expressed. Although IHC is mandatory for distinguishing HAL from metastatic HCC, no consensus exists on the immunostaining patterns for diagnosing HAL. Furthermore, the authors reported five HAL cases and described 3 immunostaining patterns between HAL and HCC: (1) CK8 and epithelial cell adhesions molecule (EpCAM) markers (HEA 125 and MOC31) are always positive in HAL (5/5 patients, 100%) and negative in HCC; (2) CK18, HepPar1, and transcription factor‐1 (TTF‐1) are always positive in HAL (5/5 patients, 100%) and HCC; and (3) cytokeratin 7 (CK7) and CEA are often positive in HAL (3/5 patients, 60%) and negative in HCC. Similar results were found by Pasricha et al. [79], who reported immunostaining patterns for six HAL cases.

### 3.5 Genetics

Genomic alterations and potential treatment targets for HACs are still unidentified due to the rarity of this tumor. A recent study by Zhao et al. [80] compared the genomic alterations of 38 HAS with 364 HCC, 437 GC, and 31 AFPGC and found that the genetic landscape of HAS was different from HCC and CGC, but that it shared mutational characteristics with AFPGC (Table 4). Furthermore, the authors found that tumor protein 53 (*TP53*) was the most commonly mutated gene in HAS (66%). *TP53* mutations were less frequent in HCC (29%), but similar in CGC (47%) and AFPGC (55%). A majority of the mutation sites were located in the *p53* DNA‐binding domain, similar to HCC, CGC, and AFPGC, but the distribution was different. These different mutation sites led to variability in the *p53* protein structure, which affected the disease outcome in these four tumors. Another study by Wang et al. [4] compared the genomic alterations of 24 HAS patients with 22 matched CGC patients and found similarly that *TP53* was the most frequently mutated gene in both groups (30.4% in HAS and 33.3% in CGC patients); however, different patterns of high‐ frequency mutations were observed (Table 4). Furthermore, Liu et al. [81] revealed that of the 25 HAS patients, the most frequently mutated genes were *TP53* (42%), mucin 19 (*MUC19*, 35%), and *TTN* (35%); however, in the 6 HACs from other organs, different gene mutations were observed (cyclin‐dependent kinase 4 [*CDK4*], keratin 34 [*KRT34*], and golgin A6 family like 3 [*GLOGA6L3*]). The previously identified driver genes in CGC, AT‐rich interactive domain 1A/1B (*ARID1A/B*) and Cadherin 1 (*CDH1*), have shown significantly lower mutation rates in HAS cases; similarly, the well‐known driver genes in HCC, *CTNNB1* and *AXIN1/2* [82].

**TABLE 4:** High‐frequency mutations between HAS, CGC, AFPGC, and HCC [4, 80].

Frequency of mutated genes (%)	HAS	CGC	AFPGC	HCC
TP53	66%	Similar (47%)	Similar (55%)	Lower (29%)
PHLDA1	13%	Lower	Lower	Lower
GMEB2	13%	Lower	Similar	Lower
CCDC15	13%	Lower	NS	Lower
PTH2	11%	Lower	NS	Lower
NFYA	8%	Lower	NS	Lower
PHKA2	8%	Similar	NS	Lower
C2orf44	8%	NS	NS	Lower
SP8	8%	Similar	Similar	Lower
IRF2BP2	8%	NS	NS	Lower
CEBPA	21.7%	0%		
RPTOR	13%	4%		
WISP3	8.7%	0.7%		
MARK1	8.7%	2.1%		
CD3EAP	8.7%	2.4%		

Abbreviations: AFPGC, AFP‐producing gastric cancer; CCDC15, coiled‐coil domain containing 15; CD3EAP, CD3e molecule epsilon‐associated protein; CEBPA, CCAAT enhancer‐binding protein alpha; CGC, common gastric cancer; GMEB2, glucocorticoid modulatory element‐binding protein 2; HAS, hepatoid adenocarcinoma of the stomach; HCC, hepatocellular carcinoma; IRF2BP2, interferon regulatory factor 2 binding protein 2; MARK1, microtubule affinity regulating kinase 1; NFYA, nuclear transcription factor Y subunit alpha; PHKA2, phosphorylase kinase regulatory subunit alpha 2; PHLDA1, pleckstrin homology‐like domain, family A, member 1; PTH2, parathyroid hormone 2; RPTOR, regulatory‐associated protein of mTOR; SP8, specificity protein 8; TP53, tumor protein 53; WISP3, WNT1‐inducible signaling pathway protein 3.

Copy number gains (CNGs) are more common in HAS compared to non‐hepatoid gastric cancers [4]. HACs with CNG at the 20q11.21‐13.12 gene locus demonstrate a more aggressive phenotype with poorer differentiation, increased vascular invasion, and a greater burden of liver metastases [83, 84]. Furthermore, reactivation of the stem cell gene SALL4, a member of the SALL gene family located at the 20q13.2 locus, has been identified as an adverse prognostic factor in several cancers [85, 86, 87]. Studies report prevalent SALL4 expression in HAS (89.0%–94.7%) compared to non‐HAS gastric cancers (10.5%–15.0%), suggesting a potential role of SALL4 in the development of HAC [4, 88]. A recent study by Ge et al. [89] assessed 31 HAS cases and found that the tumoral stromal lymphocyte programmed cell death protein‐1 (PD‐1) was positive in 58.1% and the programmed cell death ligand‐1 (PD‐L1) was positive in 45.1%. Another study revealed that of the 52 HAS cases, only three (6%) showed deoxyribonucleic acid (DNA) mismatch repair protein MutL homolog 1 (MLH1) loss, suggesting a low rate of microsatellite instability (MSI) [90].

An interesting study by Lawlor et al. [91] details the molecular characterization of 19 HAC cases from different organs and found that the primary tissue organ impacts the molecular signature. More specifically, colon HACs showed MSI, high tumor mutational burden, *ARID1A/B* gene mutations, and nuclear receptor coactivator 4 (*NCOA4‐RET*) gene fusion (2/3 cases); esophagogastric HACs revealed *TP53* mutations (2/4); biliary HAC showed loss of cyclin‐dependent kinase inhibitor 2A (*CDKN2A*, 3/4); genital HACs revealed gain of chromosome 12 (3/6); and HAL cases had serine/threonine kinase 11 (*STK11*) somatic mutations. Another review of colon HACs found that among 9 patients with reported genetic status, 8 (88.9%) had wild‐type *KRAS/NRAS*, and 1 had a mutant (11.1%) [29]. Regarding the lung, a systematic review of 51 HAL patients found that the mutation rate of epidermal growth factor receptor (*EGFR*) and anaplastic lymphoma kinase (*ALK*) was significantly lower than in non‐small cell lung cancer (NSCLC) [57, 92]. An analysis of the genetic features of four HAL cases found that *TP53* was mutated in all patients, and *CDK8*, *CDKN2A*, colony stimulating factor 1 receptor (*CSF1R*), ephrin type‐A receptor 5 (*EPHA5*), polycystic kidney and hepatic disease 1 (*PKHD1*), *SMARCA4*, and *STK11* were detected as high‐frequency mutations, with a mutation rate of 50% [93]. Furthermore, all four patients demonstrated partially positive immunostaining of both PD‐1 and PD‐L1. Currently, there are no available drug targets for *TP53* alterations. However, an interesting study by Wang et al. [94] showed that *TP53* mutations impact the expression of PD‐L1, which implies a potential sensitivity to PD‐1 and PD‐L1 checkpoint inhibitors.

## 4 Clinical Characteristics of 15 Patients Diagnosed With HAC

Using the electronic patient database at the Mayo Clinic in Rochester, Minnesota, we retrospectively searched for records of patients diagnosed with HAC from January 2004 through December 2024. Inclusion criteria were adults (> 18 years) with a diagnosis of histologically confirmed HAC with subsequent management and follow‐up at the Mayo Clinic. Patients with HAC who visited the Mayo Clinic only for a second opinion were excluded from the analysis. Descriptive statistics were used to summarize the results. Comparative data for continuous variables were reported as median (interquartile range [IQR]) or mean (standard deviation). For statistical comparison, a Chi‐square test with or without Fischer’s exact test was used for categorical variables. Survival analysis was performed with the Kaplan‐Meier method for overall mortality and log‐rank test between different treatment groups; more specifically, (a) curative surgery; (b) systemic therapy; (c) chemotherapy; (d) immunotherapy; and (e) partial or complete systemic treatment response. Furthermore, univariate Cox‐regression analysis was used to examine the association between clinical features and mortality. All reported P values were 2‐sided, and P values < 0.05 were considered statistically significant. Statistical analysis was performed using IBM SPSS Statistics, version 29.0. The study was approved by the Mayo Foundation Institutional Review Board. 

We identified 15 patients with HAC who met inclusion criteria. Most patients with HAC were males (66.7%) with a mean age at diagnosis of 66 (± 9) years, and most were White (93.3%). Table 5 lists the clinical, histological, molecular, and therapeutic characteristics of HAC patients. The most common primary tumor location was the stomach (*n* = 5, 33.3%), followed by the lung (*n* = 4, 26.7%), abdomen (*n* = 1, 6.7%), ovary (*n* = 1, 6.7%), and pancreas (*n* = 1, 6.7%). In 3 patients (20%) diagnosed with metastatic liver disease, the primary tumor site was unknown. HAC patients with unknown primary site demonstrated an increased risk for mortality (HR 5.26; 95% CI: 1.05‐26.34; *p *= 0.044), as has been reported in other cancer types with unknown primary site [95]. Among gastric primaries, the tumor was located at the gastroesophageal junction in 2 (13.3%), and in the body in 2 (13.3%). In addition, 2 patients underwent gastric surgery years prior to cancer diagnosis.

**TABLE 5:** Clinical, histological, and therapeutic characteristics of patients diagnosed with HAC.

Clinical characteristics	Frequency, *n*/*N* (%)	
Primary tumor site		
Gastric	5/15 (33.3)	
Lung	4/15 (26.7)	
Ovary	1/15 (6.7)	
Pancreas	1/15 (6.7)	
Abdomen	1/15 (6.7)	
Unknown	3/15 (20.0)	
Distant metastases at diagnosis	11/15 (73.3)	
Lymph nodes	8/15 (53.3)	
Liver	7/15 (46.7)	
Bone	3/15 (20.0)	
Intestinal	3/15 (20.0)	
Peritoneal	3/15 (20.0)	
Mesentery	2/15 (13.3)	
Lung	2/15 (13.3)	
Biliary	1/15 (6.7)	
Pancreas	1/15 (6.7)	
Adrenal	1/15 (6.7)	
Regional lymph node metastases at diagnosis	3/15 (20.0)	
**Therapeutic management**		**Survival (months)**
Curative surgery	5/15 (33.3)	Mean 24 (±17)
Systemic therapy	11/15 (73.3)	
Systemic chemotherapy^a^	10/14 (71.4)	Median 15 (3–114)
Platinum‐based	9/14 (64.3)	
5‐Fluorouracil	4/14 (28.6)	
Taxanes	4/14 (28.6)	
Topoisomerase inhibitors	3/14 (21.4)	
Pemetrexed	2/14 (14.3)	
Gemcitabine	1/14 (7.1)	
Evofosfamide	1/14 (7.1)	
Targeted therapy	8/14 (57.1)	Mean 18 (±11)
Vascular endothelial growth factor inhibitors	5/14 (35.7)	
Receptor tyrosine kinase inhibitors	1/14 (7.1)	
Navitoclax	1/14 (7.1)	
Sorafenib	1/14 (7.1)	
Immunotherapy	6/14 (42.9)	Mean 18 (±13)
Radiation	5/15 (33.3)	Mean 14 (±7)
Local therapy	2/15 (13.3)	
Radio‐embolization of liver lesion	1/15 (6.7)	
Radiofrequency ablation of liver lesion	1/15 (6.7)	
Laboratory test results prior to diagnosis		
Anemia	7/15 (46.7)	
Elevated liver function tests	9/13 (69.2)	
Carcinoembryonic antigen (CEA)	8/12 (66.7)	
Alpha‐fetoprotein (AFP)	5/12 (41.7)	
Carbohydrate antigen (CA) 19‐9	3/10 (30.0)	
Immunohistochemistry markers		
Hep‐Par 1	9/12 (75.0)	
Cytokeratin 7 (CK7)	5/9 (55.6)	
Cytokeratin 20 (CK20)	2/7 (28.6)	
Cytokeratin AE1/AE3 (CK AE1/AE3)	1/6 (16.7)	
Epithelial specific antigen MOC‐31	4/4 (100.0)	
Polyclonal carcinoembryonic antigen (CEA)	4/5 (80.0)	
Mucin production	2/4 (50.0)	
Caudal type homeobox transcription factor‐2	1/8 (12.5)	
Glypican‐3	4/6 (66.7)	
Albumin in situ hybridization	2/5 (40.0)	
Arginase‐1	3/7 (42.9)	
Thyroid transcription factor‐1	2/9 (22.2)	
Paired box‐8 transcription factor	1/5 (20.0)	
Genetic testing		
Microsatellite instability (MSI)	0/8 (0.0)	
Tumor protein 53 (*TP53*)	5/8 (62.5)	
Overall survival	6/15 (40.0)	
Survival at 1‐year	8/15 (53.3)	
Survival at 3‐years	3/15 (20.0)	
Survival at 5‐years	1/15 (6.7)	

Abbreviation: HAC, hepatoid adenocarcinoma.

^a^ Systemic chemotherapy drug details were not available for one patient.

The majority of patients (73.3%) presented with distant metastases at the first diagnosis of HAC. Almost half of HAC patients (46.7%) presented with a liver mass, and the initial diagnosis of HAC in these patients was made by biopsy of the liver mass. About 20% had regional lymph node metastases at diagnosis, and only one patient (6.7%) had no metastases. Only one patient with liver metastasis had underlying cirrhosis; as a result, a medical history lacking cirrhosis or infection with chronic viral hepatitis may favor the diagnosis of HAC. Most patients (86.7%) experienced symptoms prior to diagnosis. HAC was an incidental finding on imaging studies in three patients (20%). Diagnosis at an early stage was mainly made incidentally on imaging studies performed for other medical conditions. Acute symptoms developed in 26.7% of patients and chronic symptoms (> 30 days) in 73.3%. The majority of HAC patients manifested gastrointestinal symptoms (66.7%), followed by general symptoms (40%), respiratory‐related (13.3%), joint‐related (20%), and gynecologic (6.7%). The most common symptoms in our cohort were abdominal pain and weight loss. Complications at first diagnosis were observed in 60% of HAC patients, including thromboembolic events (33.3%), bone fracture due to lytic lesions (6.7%), and ascites (6.7%).

A significant proportion of patients (60%) had elevated liver enzymes at diagnosis, and almost half had anemia (46.7%). CEA was increased in 53.3% of patients, AFP in 33.3%, and CA 19‐9 in 20%. Elevated AFP levels were observed in 3 out of 4 gastric HACs (75%) with available AFP levels, of whom *n* = 2 had serum AFP levels > 500 ng/mL. Increased AFP levels were also observed in *n* = 1 patient with ovarian HAC, and *n* = 1 with unknown primary HAC location, all of which had distant metastases at the time of diagnosis, including the gastric cases. However, none of our patients with lung HAC had increased serum AFP levels (3 out of 3 with available AFP levels, 0%). Interestingly, only one of these patients was diagnosed with distant metastasis at the time of diagnosis. Compared to other studies, the proportion of gastric HACs with elevated serum AFP is slightly lower, while the proportion of lung HACs is significantly lower, as demonstrated in Table 1. Further analysis revealed that elevated serum AFP levels were not associated with poorer survival in our cohort (HR 0.99; 95% CI: 0.20‐5.01; *p* = 0.991); however, our small sample size might underestimate this association. Serum AFP levels decreased in all patients with available AFP levels following treatment (4 out of 4 patients, 100%); however, these patients experienced a significant increase in AFP levels with disease progression.

**FIGURE 1:**
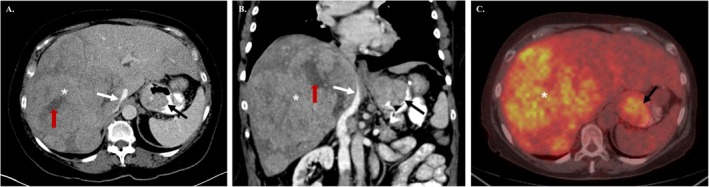
Gastric hepatoid adenocarcinoma with liver metastases in a 65‐year‐old‐female with prior history of Roux‐en‐Y gastric bypass surgery. Contrast enhanced axial CT image (A) and coronal reformatted image (B) showing a large lobulated and heterogeneously enhancing metastases in right lobe liver (*) with mass effect on inferior vena cava (white arrows) and areas of necrosis (red arrows). The primary HAC is identified in the gastric pouch (black arrows). Fused fluorodeoxyglucose (FDG) positron emission tomography (PET)‐CT image (C) showing increased tracer uptake in the hepatic metastasis (*) and in the primary gastric tumor (black arrow).

**FIGURE 2:**
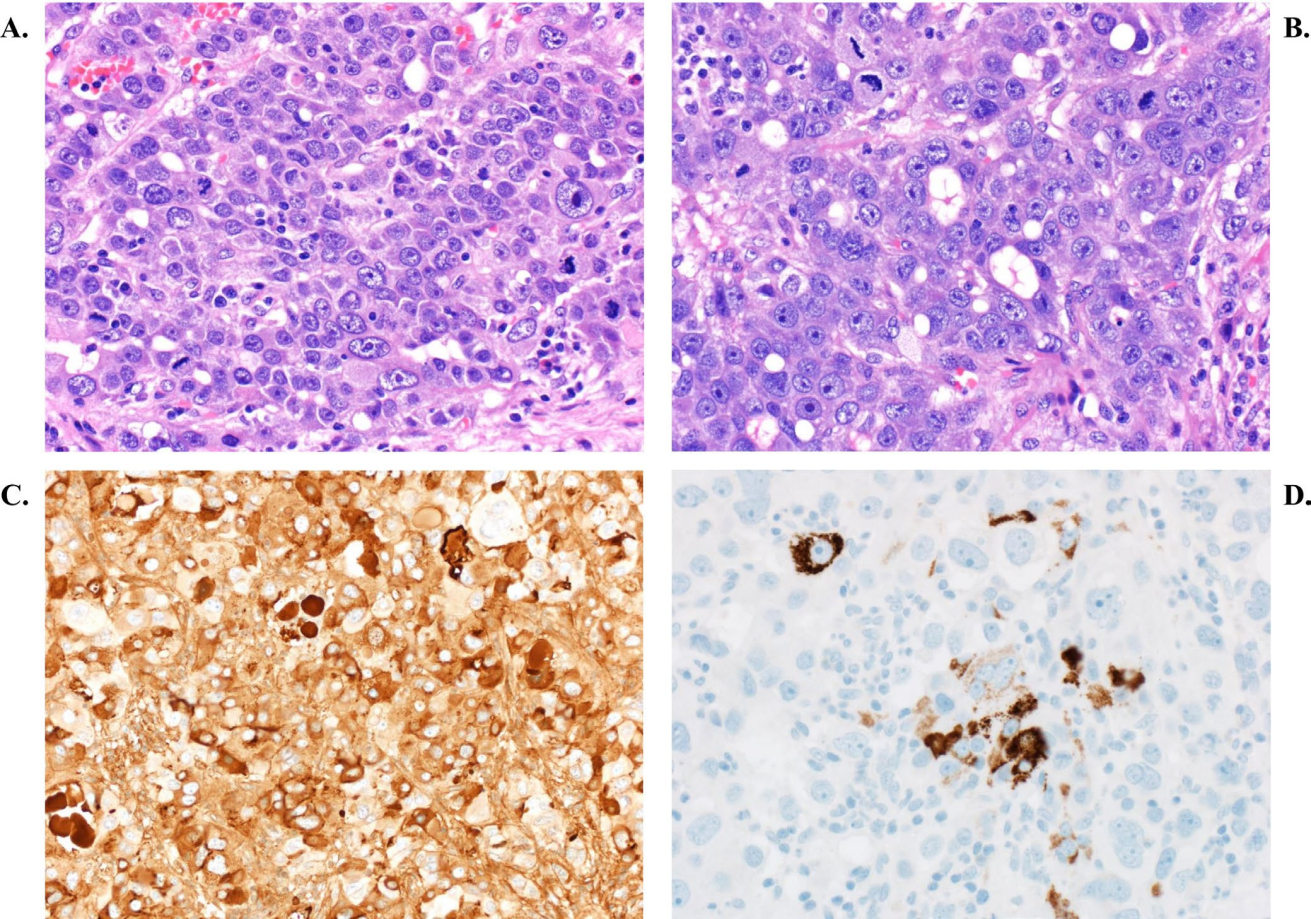
(A) Hepatoid adenocarcinoma from the stomach. Solid areas of growth are seen. (B) Gland formation was also evident. (C) The tumor cells show diffuse AFP expression. (D) The tumor cells showed patchy HepPar1 staining.

Similar to HCC, liver metastases of HAC demonstrated arterial hyperenhancement with late washout on CT imaging (Figure 1). On histology, features of HAC were identified by gland formation and mucin production (Figure 2A and 2B). IHC analysis revealed positive AFP staining (Figure 2C). Compared to other studies, we observed a higher frequency of Hep‐Par 1 positivity on IHC analysis (9 out of 12 HAC patients with IHC staining, 75%, Figure 2D). None of the HACs was human epidermal growth factor receptor 2 (HER2) positive (0 out of 6). MSI was also negative in all tested HAC patients (0 out of 9), which results in lower efficacy of systemic therapy. *TP53* gene was the most frequently mutated gene (5 out of 8, 62.5%) in our cohort, similarly to other studies, where the frequency varied from 30.4% to 66% [4, 80, 81]. In other adenocarcinomas, a lower frequency of *TP53* mutations was observed (47% in CGC, 55% in AFPGC, and 29% in HCC, as shown in Table 4). Mutations in *TP53* leads to inactivation of the tumor suppressor protein *p53* and genomic instability, which results in extensive chromosomal abnormalities, increased rates of DNA alterations, genomic instability, and ultimately cancer progression [96, 97]. The aggressive biological behavior, poor prognosis, and lower survival rates of HAC may be influenced by the disrupted apoptosis and DNA repair pathways that are facilitated by *TP53* mutations. 

PD‐L1 expression showed a positive score in 3 out of 6 patients (50%). Interestingly, all patients with PD‐L1 positive expression (*n* = 3) had TP53 mutations present in the genetic testing. Of these, only *n* = 1 patient received immunotherapy with partial/complete response, while of the *n* = 2 remaining patients, one died within 3 months of HAC diagnosis without receiving any treatment, and the other underwent curative gastrectomy without further treatment with systemic chemotherapy. Studies have shown a positive correlation between *TP53* gene mutations and higher PD‐L1 expression in several cancers, mainly NSCLC, suggesting potential benefit from combination therapies, such as PD‐L1 inhibitors and regimens currently under development which target p53 [98, 99]. Furthermore, myelocytomatosis oncogene (MYC) mutations were identified in 4 out of 8 patients (50%) in our cohort. Additional mutated genes found in our cohort of HAC patients are summarized in Supplementary Table S1, and systemic treatment regimens used in our cohort as well as their dosage and duration are demonstrated in Supplementary Table S2.

Five patients had curative surgical resection, *n* = 11 (73.3%) received systemic therapy [*n* = 10 (66.7%) chemotherapy, *n* = 8 (57.1%) targeted therapy, and *n* = 6 (40%) immunotherapy], and *n* = 5 (33.3%) underwent radiation. Of the *n* = 10 patients with available systemic treatment regimens in our cohort, half (*n* = 5) received anti‐PD‐L1 therapy, of whom *n* = 4 had available data about treatment response. Most of these patients (*n* = 3) showed partial or complete response to anti‐PD‐L1 treatment, while only *n* = 1 patient experienced treatment failure. The mixed efficacy of immunotherapy might be due to heterogeneity of the tumor microenvironment, cancer cell adaptation leading to drug resistance, insufficient T‐cell infiltration, and lack of neoantigens [100]. Future research directions are required for precision approaches to address the current challenges with immunotherapy, including next‐generation therapies, personalized neoantigen vaccines, predictive biomarkers for optimal patient selection and biomarker‐driven trials [100]. Regarding local therapy, transarterial chemoembolization (TACE) in combination with systemic chemotherapy was performed in *n* = 1 patient with liver metastases at HAC diagnosis and unknown primary site; however, the short‐term follow‐up imaging did not reveal any response. Furthermore, radiofrequency ablation (RFA) in combination with systemic chemotherapy was conducted in *n* = 1 patient with liver metastasis and gastric HAC, and disease recurrence was identified four months later. However, no surgery was performed on any of the patients who underwent TACE or RFA.

**FIGURE 3:**
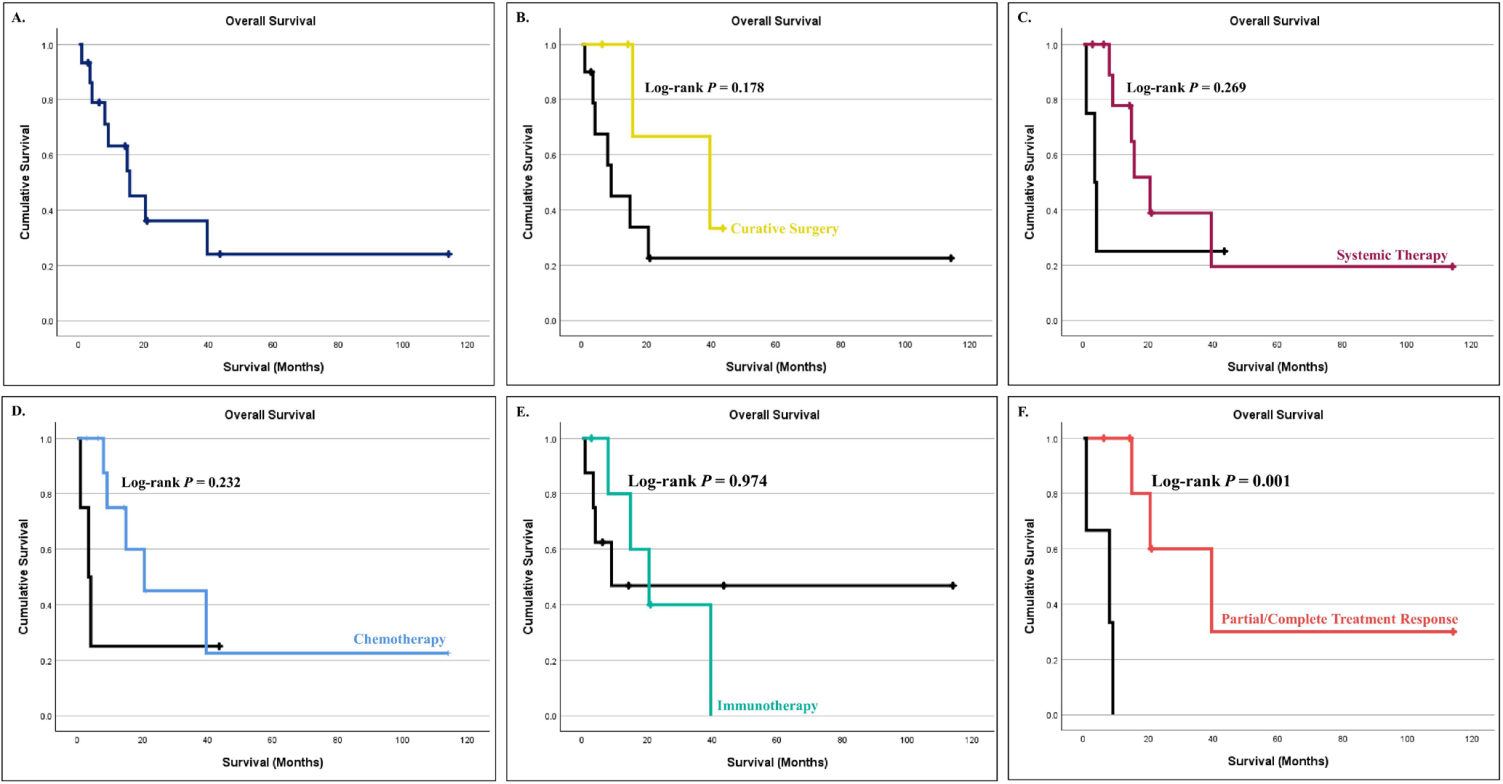
Kaplan‐Meier survival curves for overall survival of: (A) patients diagnosed with HAC (dark blue line), (B) those who underwent curative surgery (yellow line), (C) those who received systemic therapy (purple line), (D) those who received chemotherapy (light blue line), (E) those who received immunotherapy (green line), and (F) those with partial or complete systemic treatment response (red line).

Median survival for HAC patients was 14 (1‐114) months (Figure 3A). No survival benefit was observed between groups treated with curative surgery (*P* = 0.178, Figure 3B), systemic therapy (*p*= 0.269, Figure 3C), chemotherapy (*p* = 0.232, Figure 3D), or immunotherapy (*p* = 0.974, Figure 3E). However, only a few patients received immunotherapy (6 out of 14, 42.9%), which suggests that the numbers are too small to draw a conclusion about its efficacy in treating HAC. Patients who achieved partial or complete response to systemic therapy had higher survival rates at 1 year compared to those who failed treatment (85.7% vs 0% in treatment failure, *p* = 0.033), as well as overall survival (42.9% vs 100%, *p* = 0.001, Figure 3F).

Nevertheless, the small sample size of our study has limitations, such as reduced statistical power to detect real treatment effects, imprecise interpretation of variations in treatment responses across different patient subgroups, increased risk of random errors and biased results, and inability to generalize the results to the larger population. Thus, larger multi‐center cohort studies are required to validate and confirm these findings.

## 5 Treatment and Prognosis

There are no specific guidelines from the National Comprehensive Cancer Network (NCCN) or the European Society for Medical Oncology (ESMO) for the treatment of HAC, as it represents a rare type of adenocarcinoma with varied primary sites. Management follows the broader, general cancer guidelines for the primary organ site with an emphasis on surgical resection and palliative care as well as advanced treatment options [101, 102]. Surgical resection for early‐stage tumors is considered the only curative therapy for gastric HAC [5]. Surgery is commonly done for palliation as well and may positively impact prognosis [14, 103, 104, 105]. Optimal adjuvant or neoadjuvant therapies are not well‐defined. A recent systematic review of response to chemotherapeutic regimens employed in the management of metastatic HAC included 18 case reports but overall included only 22 patients with metastatic HAC with gastric (*n* = 11) or other primary locations (*n* = 7), including lung, colon, pancreas, and mediastinum [106]. Partial or complete response to chemotherapy was noted in 11 cases, a majority of whom (81%) had received a cisplatin‐based regimen. Decreased response and survival were noted in patients treated with irinotecan, oxaliplatin, 5‐fluorouracil, or gemcitabine.

A recent comprehensive analysis of HAL cases demonstrated that the combination of paclitaxel and platinum‐based chemotherapy was efficient as first‐line treatment, with the integration of immunotherapy and targeted therapies showing potential benefits [18]. However, the response of HAC to molecular targeted therapy has not yet been well described, although a positive response to the multikinase inhibitor sorafenib has been reported [107, 108]. Early stage HACs often respond well to radical surgical resection, leading to longer survival. For advanced stages, cisplatin‐based chemotherapy, immunotherapy, targeted therapy, and radiotherapy show promise for managing symptoms and prolonging survival, though prognosis remains poor. Compared to conventional adenocarcinomas, HACs are generally associated with a worse prognosis and are more aggressive with a predisposition for diagnosis at advanced disease stages, making them less responsive to standard treatments, which further contributes to higher mortality rates [18, 109, 110].

HAC is associated with a poor prognosis perhaps due to its propensity for vascular invasion and hematogenous spread [11, 69, 111]. A retrospective study examined prognostic factors in 20 patients with gastric HAC, 17 (85%) of whom had metastatic disease at the time of diagnosis [104]. Survival ranged from 2 to 99 months with a median survival time of 12 months and a 3‐year survival rate of 17.2%, similar to our study. This study revealed improved survival among those patients treated with surgery, either radical or palliative, and adjuvant chemotherapy. Advanced stage at diagnosis predicted worse survival. Small case reports described the use of TACE along with systemic chemotherapy in patients with HAS and metastatic liver lesions; however, recurrence was observed with development of new liver metastases [112]. Another case report demonstrated good response to repeated sessions of microwave RFA in small metastatic lesions in the lung and liver in combination with systemic chemotherapy and surgery, with significant reduction in AFP levels and no recurrence of HAS at 19 months [113]. These results suggest that TACE and RFA may serve as a local therapy option for metastatic liver lesions of HAS when used in combination with systemic chemotherapy and radical surgery.

A few recent studies describe therapeutic strategies based on specific cancer gene mutations that are observed in HACs. For example, studies have shown that *ARID1A*‐mutated cancers might benefit from immune checkpoint blockage, mammalian target of rapamycin (mTOR) inhibitors, targeted therapies with enhancer of zeste homolog 2 (EZH2) inhibitors, histone deacetylases, ataxia telangiectasia and Rad3‐Related (ATR) inhibitors, and/or Poly (ADP‐ribose) Polymerase (PARP) inhibitors. On the other hand, *ARID1A* mutations may also mediate resistance to platinum chemotherapy and estrogen degraders/modulators [114, 115]. As for HER2‐positive cancers, targeted therapies are being used to block HER2 receptor or signaling pathways, and cause tumor cell death. The HER2‐targeted drugs include monoclonal antibodies (such as trastuzumab, pertuzumab, zanidatamab), tyrosine kinase inhibitors (TKIs, such as lapatinib, tucatinib), and antibody‐drug conjugates (ADCs, such as trastuzumab deruxtecan, trastuzumab emtansine), often combined with chemotherapy [116]. Novel targeted therapies have emerged for tumors with *TP53* mutations; however, currently there are no approved drugs. (Ongoing research focuses on 1) restoring normal p53 function and developing p53 reactivators, such as mouse double minute 2 (MDM2) degraders, p53 stabilizers, and gene therapies targeting (*TP53*; 2) utilizing MDM2 inhibitors; and 3 employing immunotherapy to target mutant p53 [117].

## 6 Conclusion

HAC is a rare tumor with poor prognosis and aggressive biological behavior. HAC is usually diagnosed at an advanced stage with distant metastases. In patients diagnosed with liver lesions that have similar radiologic and histologic features to HCC, particularly in the absence of underlying chronic liver disease, further evaluation should be performed to rule out HAC. Communication between medical subspecialties is important to avoid misdiagnosis and prevent further disease progression. In order to optimize management of patients diagnosed with HAC, molecular profiling and, more importantly, *TP53* status and PD‐L1 expression, should be routinely performed, as these patients may benefit from immunotherapy and targeted therapies. Due to its rarity, there is a lack of standardized treatment protocols, and the pathogenesis remains largely unknown. A deeper understanding of the molecular mechanisms driving carcinogenesis and the occurrence of HAC is warranted.

## Author Contributions

**Christina Liava:** conceptualization (equal), data curation (equal), formal analysis (equal), investigation (equal), methodology (equal), resources (equal), software (equal), validation (equal), visualization (equal), writing – original draft (equal), writing – review and editing (equal). **Sudhakar Venkatesh:** data curation (supporting), formal analysis (supporting), investigation (supporting), resources (equal), validation (equal), visualization (equal), writing – original draft (equal), writing – review and editing (equal). **Michael S. Torbenson:** data curation (supporting), formal analysis (supporting), investigation (supporting), resources (equal), validation (equal), writing – original draft (equal), writing – review and editing (equal). **Patrick S. Kamath:** conceptualization (lead), methodology (lead), project administration (lead), supervision (lead), validation (lead), visualization (lead), writing – original draft (equal), writing – review and editing (lead). **Moira Hilscher:** conceptualization (lead), project administration (lead), supervision (lead), validation (equal), visualization (equal), writing – original draft (equal), writing – review and editing (lead).

## Funding

The authors have nothing to report.

## Ethics Statement

The study was approved by the Mayo Foundation Institutional Review Board (IRB application no.: 24‐012242).

## Conflicts of Interest

The authors declare no conflicts of interest.

## Supporting information


**Table S1:** Supporting Information.

**Table S2:** Supporting Information.

## Data Availability

Data available on request. The data underlying this article will be shared on reasonable request to the corresponding author.
