# Supplementary material for: A Clinical Guidance for the Management of Patients With Hepatoid Adenocarcinoma and A Case Series
Source: Cancer Med. 2026 Feb 5;15(2):e71398. doi: 10.1002/cam4.71398 (PMC12873860; doi:10.1002/cam4.71398)
Supplement: Supplementary file 1 — Table S1: Supporting Information. [file CAM4-15-e71398-s001.docx]

**SUPPLEMENTARY TABLES**

**Supplementary Table 1.** Frequency of Mutated Genes Observed in the 15 HAC Patients

| Mutated Genes | Frequency of Mutated Genes, *n/N (%)* |
| --- | --- |
| TP53 | 5/8 (62.5) |
| MYC | 4/8 (50.0) |
| BRCA1 or BRCA2 | 4/8 (50.0) |
| NRAS | 2/8 (25.0) |
| CCNE1 | 1/8 (12.5) |
| ERBB2 or ERBB3 | 1/8 (12.5) |
| ARID1A | 1/8 (12.5) |
| ROS1 | 1/8 (12.5) |
| GABRA6 | 1/8 (12.5) |
| KDM6A | 1/8 (12.5) |
| SRC | 1/8 (12.5) |
| TSC1 | 1/8 (12.5) |
| TYRO3 | 1/8 (12.5) |
| WHSC1 | 1/8 (12.5) |
| ZRSR2 | 1/8 (12.5) |
| AKT2 | 1/8 (12.5) |
| CRKL | 1/8 (12.5) |
| TET2 | 1/8 (12.5) |
| BCOR | 1/8 (12.5) |
| BRD4 | 1/8 (12.5) |
| KMT2C | 1/8 (12.5) |
| NOTCH1 | 1/8 (12.5) |
| ALK | 1/8 (12.5) |
| SMARCA4 | 1/8 (12.5) |
| RAD21 | 1/8 (12.5) |
| PBRM1 | 1/8 (12.5) |
| CDK6 | 1/8 (12.5) |
| CPS1 | 1/8 (12.5) |
| MCL1 | 1/8 (12.5) |
| EGFR | 1/8 (12.5) |
| CDKN2A | 1/8 (12.5) |
| KMT2D | 1/8 (12.5) |

*HAC: Hepatoid Adenocarcinoma; TP53: Tumor Protein 53; BRCA: Breast Cancer;* *CCNE1: Cyclin E1; ERBB: Erythroblastic Oncogene B; ARID1A: AT-rich Interactive Domain 1A; GABRA6: Gamma-Aminobutyric Acid Receptor Subunit Alpha-6; KDM6A: Lysine (K)-Specific Demethylase 6A; WHSC1: Wolf-Hirschhorn Syndrome Candidate 1; ZRSR2: Zinc Finger (CCCH type) RNA Binding Motif and Serine/Arginine Rich 2; CRKL: CRK Like Proto-oncogene; TET2: Tet Methylcytosine Dioxygenase 2; BCOR: BCL6 Corepressor; BRD4: Bromodomain-Containing Protein 4; KMT2C: Lysine N-Methyltransferase 2C; NOTCH1: Neurogenic Locus Notch Homolog Protein 1; ALK: Anaplastic Lymphoma Kinase; PBRM1: Polybromo 1; CDK6: Cyclin-Dependent Kinase 6; CPS1: Carbamoyl-Phosphate Synthase 1; EGFR: Epidermal Growth Factor Receptor; CDKN2A: Cyclin-Dependent Kinase Inhibitor 2A; KMT2D: Lysine (K)-Specific Methyltransferase 2D.*
