# Supplementary material for: A Clinical Guidance for the Management of Patients With Hepatoid Adenocarcinoma and A Case Series
Source: Cancer Med. 2026 Feb 5;15(2):e71398. doi: 10.1002/cam4.71398 (PMC12873860; doi:10.1002/cam4.71398)
Supplement: Supplementary file 2 — Table S2: Supporting Information. [file CAM4-15-e71398-s002.docx]

**Supplementary Table 2.** Systemic Treatment Regimens, Dosages, and Therapy Duration

| First-Line Treatment | Dosage | Duration-Months | Second-Line Treatment | Dosage | Duration-Months | Third-Line Treatment | Dosage | Duration-Months | Fourth-Line Treatment | Dosage | Duration-Months |
| --- | --- | --- | --- | --- | --- | --- | --- | --- | --- | --- | --- |
| Oxaliplatin  Leucovorin  Fluorouracil | 85-100 mg/m^2^  400 mg/m^2^  400 mg/m^2^ | 7 | Irinotecan  Leucovorin  Fluorouracil | 180 mg/m^2^  400 mg/m^2^  400 mg/m^2^ | 3 |  |  |  |  |  |  |
| Pemetrexed  Carboplatin  Pembrolizumab | N/A  N/A  N/A | 3 |  |  |  |  |  |  |  |  |  |
| Gemcitabine  Cisplatin | N/A  N/A | 3 | Atezolizumab  Bevacizumab | N/A  N/A | 5 |  |  |  |  |  |  |
| Oxaliplatin  Leucovorin  Fluorouracil  Docetaxel | N/A  N/A  N/A  N/A | 3 | Pembrolizumab | N/A | 5 | Pembrolizumab  Ramucirumab  Paclitaxel | N/A  N/A  N/A | 16 | Irinotecan  Leucovorin  Fluorouracil  Pembrolizumab  Ramucirumab | 180 mg/m^2^  400 mg/m^2^  400 mg/m^2^  N/A  N/A | 13 |
| Docetaxel  Leucovorin  Fluorouracil | 40 mg/m^2^/day  400 mg/m^2^  400 mg/m^2^ | 5 | Every 48 hours  Fluorouracil | 2000mg/m^2^ | 94 |  |  |  |  |  |  |
| Sorafenib  Navitoclax | N/A  N/A | 12 | Ipilimumab  Evofosfamide | N/A  N/A | 9 |  |  |  |  |  |  |
| Oxaliplatin  Leucovorin  Fluorouracil  Bevacizumab | N/A  N/A  N/A  N/A | 9 | Carboplatin  Pemetrexed  Bevacizumab | N/A  N/A  N/A | 2 |  |  |  |  |  |  |
| Carboplatin  Paclitaxel | N/A  N/A | 2 | Atezolizumab  Carboplatin  Paclitaxel  Bevacizumab | N/A  N/A  N/A  N/A | 2 | Atezolizumab | N/A | 8 |  |  |  |
| Cisplatin  Etoposide | N/A  N/A | 4 | Atezolizumab  Bevacizumab | N/A  N/A | 9 |  |  |  |  |  |  |
| Carboplatin  Paclitaxel | N/A  N/A | 3 | Lenvatinib | N/A | 6 | Atezolizumab  Bevacizumab | N/A  N/A | 12 |  |  |  |

*mg/m^2^: milligram per square meter; N/A: Not Available.*
